# On the Comparative Value of the Diagnostic Signs of Pneumonia

**Published:** 1851-03

**Authors:** 


*On the comparative value of the Diagnostic Signs of Pneumonia.* By Professor Alison.—The following valuable observations occur in the course of a clinical lecture on some cases of pleurisy and pneumonia:

As to the special indications of peripneumony, whether with or without attending pleurisy, I think we shall find the most important and practically useful to be that given by the *sputa*—i. e., the appearance of tenacious and at first translucent sputa, thoroughly *stained* with blood, and thereby acquiring a considerable variety of colors in different cases, the reasons of which variety I do not pretend to know,—rusty, pink, purple, dark yellow, or greenish,—all to be regarded as inflammatory; but the darker colored, or more opaque, being generally the indication of the more advanced stage of inflammation. The *crepitous râle* is in some cases very distinct and satisfactory, but in many it is unperceived, or if perceived is faint and of short duration in the early stage of the disease, while it may be of long standing in the advanced stage, and during the convalescence; and in some it is not so easily distinguished from the somewhat coarser sound of the subcrepitous râle, which may depend on conditions in the lungs, or minute branches of the bronchial, very different from inflammation. On these accounts, this symptom has never seemed to me to realize the very natural hopes of Laennec, as to its practical usefulness in detecting pneumonia, when not otherwise indicated.

The other indications that we have of peripneumony by auscultation and percussion—dullness on percussion, bronchial sound, respiration, or its complete suppression, and imperfect expansion of the affected side—are rather, in fact, what may be expected, and can hardly be distinguished from the effects of attendant pleurisy, so frequently complicating that disease.

In regard to all those physical or local symptoms of peripneumony, this observation, I am sure, we shall find practically important, that although we may rely much on them, particularly when grouped together, and watched in their progress, as indicating the *nature* of the disease, we cannot rely on them at all as indicating its *intensity*, its *probable tendency*, or the *extent* to which antiphlogistic remedies during it are demanded, or can be safely employed.

Take, for example, the pneumonic sputa,—they may be seen in full perfection in cases which are partial and slight, or which are attended with extreme debility, demanding stimulants; and may be absent in cases which are extensive and urgent, and will bear full evacuation. I remember having two young men—both previously in good health, and seen early—under my care at the same time, in pneumonia; in one of whom we had the translucent sputa almost from the first, exactly resembling red-current jelly, but he was so feeble that he became faint the first time I set him up to examine his chest; and although I thought it right to take some blood from him, he did not lose ten ounces, generally or locally. In the other, we never saw any tinge of blood in the sputa, but the other symptoms were well marked and urgent, and he bore four full general bleedings perfectly well; the blood was sizy, and the symptoms were relieved, as usual, after each bleeding. Both these patients recovered perfectly well; and judging by the progress of the cases,—especially as regards the state of the vital functions, the pulse, and breathing,—we had no reason to regret either that the first was so little, or the second so fully bled; but if we had trusted to the sputa as indicating the nature and intensity of the disease, we should have thought that the first demanded the full antiphlogistic treatment, and the last little or none. And I have seen several in which the peripneumonic sputa were peculiarly well marked, which I have thought it necessary to treat, from the time I first saw them, (late in the disease), by stimuli only, and which have recovered remarkably well.

Again, as to the crepitous râle, I have seen many cases which seemed to me well marked by all the other symptoms, and which have done well, generally, under the antiphlogistic treatment, in which I never could detect that symptom; and others, in which I was quite satisfied of its existence and extension through the lungs, while the other symptoms have either continued so mild or been of such a nature that no active antiphlogistic practice has appeared to be demanded or justified by that extension, and the whole symptoms have subsided without them. Particularly since I read the statements of M. Louis, as to the extension of the crepitous râle through the lungs, notwithstanding full bleeding, by which the pulse had been brought down,—from which he inferred that the idea of the power of bloodletting over inflammation had been a mistake,—I have attended to this point, and satisfied myself that he was quite correct as to the fact, but wrong as to the inference,—I mean as a general principle; because, in these circumstances, when the pulse and breathing have decidedly improved, although the râle may indicate that the inflammation continues and extends for a time, we may be very confident that it will subside without further treatment. The reason, probably, is, that the inflammatory exudation, although extending, is modified in character, and so attenuated as to be easily reabsorbed,—so that no complete obstruction, or permanent disorganization of the pulmonary substance, to any injurious extent, is effected. In these circumstances, all that is required is, to watch the patient carefully and revert immediately to the bleeding, or other antiphlogistic treatment, if the breathing becomes again more oppressed, and the pulse more frequent and *firmer;* under the conviction, given by experience, that the crepitous râle extending through the lungs in a case of recent illness, and in connection with a frequent and full pulse, and a labored state of the breathing, implies much danger, which may be averted by such treatment; but that the same râle extending through the lungs, in connection with a more natural pulse, and an easy state of the breathing, or with dyspnœa, but a feeble or soft pulse, and damp, livid skin, either implies no danger, and demands no active treatment, or is to be counteracted by very different remedies. And I may add, that this is exactly the line of conduct which our predecessors would have advised in those circumstances, before the crepitous râle was heard of, although they could not have spoken with so much precision as we can, of the nature of the changes then going on in the lungs.—*Edinburgh Monthly Journal*, from *Ranking's Abstract.*

